# Vitamin D Deficiency in Unselected Patients from Swiss Primary Care: A Cross-Sectional Study in Two Seasons

**DOI:** 10.1371/journal.pone.0138613

**Published:** 2015-09-15

**Authors:** Christoph Merlo, Michael Trummler, Stefan Essig, Andreas Zeller

**Affiliations:** 1 Institute of Primary and Community Care, Schwanenplatz 7, 6004, Luzern, Switzerland; 2 Laboratories Bioanalytica, Maihofstrasse 95A, 6006, Luzern, Switzerland; 3 Swiss Paraplegic Research, Guido A. Zäch Strasse 4, 6207, Nottwil, Switzerland; 4 Center for Primary Health Care, University of Basel, Rheinstrasse 26, 4410, Liestal, Switzerland; University of Alabama at Birmingham, UNITED STATES

## Abstract

**Background:**

As published data on 25-hydroxy-cholecalciferol (25(OH)D) deficiency in primary care settings is scarce, we assessed the prevalence of hypovitaminosis D, potential associations with clinical symptoms, body mass index, age, Vitamin D intake, and skin type in unselected patients from primary care, and the extent of seasonal variations of serum 25(OH)D concentrations.

**Methodology/Principal Findings:**

25(OH)D was measured at the end of summer and/or winter in 1682 consecutive patients from primary care using an enzyme-linked immunosorbant assay. Clinical symptoms were assessed by self-report (visual analogue scale 0 to 10), and vitamin D deficiency was defined as 25(OH)D concentrations < 50 nmol/l. 25(OH)D deficiency was present in 995 (59.2%) patients. 25(OH)D deficient patients reported more intense muscle weakness (visual analogue scale 2.7, 95% confidence interval 2.5 to 2.9) and had a higher body mass index (25.9kg/m^2^, 25.5 to 26.2) than non-deficient patients (2.5, 2.3 to 2.7; and 24.2, 23.9 to 24.5, respectively). 25(OH)D concentrations also weakly correlated with muscle weakness (Spearman’s rho -0.059, 95% confidence interval -0.107 to -0.011) and body mass index (-0.156, -0.202 to -0.108). Self-reported musculoskeletal pain, fatigue, and age were not associated with deficiency, nor with concentrations. Mean 25(OH)D concentrations in patients with vitamin D containing medication were higher (60.6 ± 22.2 nmol/l) than in patients without medication (44.8 ± 19.2 nmol/l, p < 0.0001) but still below the targeted level of 75 nmol/l. Summer and winter 25(OH)D concentrations differed (53.4 ± 19.9 vs. 41.6 ± 19.3nmol/l, p < 0.0001), which was confirmed in a subgroup of 93 patients who were tested in both seasons (p = 0.01).

**Conclusion/Significance:**

Nearly 60% of unselected patients from primary care met the criteria for 25(OH)D deficiency. Self-reported muscle weakness and high body mass index were associated with lower 25(OH)D levels. As expected 25(OH)D concentrations were lower in winter compared to summer.

## Introduction

Low serum levels of 25-hydroxy vitamin D3 (25(OH)D) have been increasingly recognised as a major health problem of public concern [1]. The essential role of 25(OH)D in bone metabolism and muscular function is comprehensively documented [2–4]. Patients with severe 25(OH)D deficiency (serum concentrations of 25(OH)D below 25 nmol/l) are at overt risk of osteomalacia and osteoporosis leading to falls and osteoporotic fractures, particularly in elderly people [5]. Vitamin D deficiency has also been related to nonspecific symptoms such as muscular weakness, fatigue, and musculoskeletal pain [4]. In the past several years, scientific attention has additionally turned to non-skeletal effects of 25(OH)D [5, 6]. Evidence from numerous studies suggests an association between 25(OH)D deficiency and several chronic conditions such as cardiovascular disease, diabetes mellitus, cancer, depression, and immune dysfunction [7–10] as well as an increased all-cause mortality risk [9]. As a consequence, large interventional studies to assess the potential beneficial effects of 25(OH)D supplementation on several non-skeletal health conditions are currently being conducted, e.g. the DO-HEALTH study in Europe (www.do-health.eu) and the VITAL study in the US (www.vitalstudy.org).

The main risk factor for the development of 25(OH)D deficiency is insufficient exposure to sunlight since the main source of 25(OH)D is the endogenous production in the skin by solar ultraviolet light B (UVB) [11]. Anything that diminishes the transmission of solar UVB radiation to the earth’s surface (e.g. geographical latitude) or anything that interferes with the penetration of UVB radiation into the skin (e.g. degree of skin pigmentation, sunscreens, or practice of purdah) will affect the cutaneous synthesis of 25(OH)D [12]. Only few foods including oily fish such as salmon, mackerel, and herring naturally contain relevant amounts of 25(OH)D, and therefore, dietary intake is considered to be a minor source (about 10%) of 25(OH)D supply [4]. Aging is also considered as a possible factor affecting the production of 25(OH) and the development of 25(OH)D deficiency [13]. As the body ages the capacity of the human skin to produce 25(OH)D may decline because the amount of 7-dehydrocholesterol—the precursor of 25(OH)—in skin cells decreases [14].

Furthermore, a consistent association between increasing body mass index (BMI) and lower serum 25(OH)D concentrations has been reported in the literature [15, 16] Because 25(OH)D is fat soluble, it is readily taken up by fat cells. Due to the sequestration of 25(OH)D by the large body fat pool, obesity is believed to be associated with 25(OH)D deficiency [17]. Musculoskeletal pain, general muscular weakness, and fatigue are common symptoms particularly in general practice and have numerous and often non-specific aetiologies. Hypovitaminosis D may be one cause of such symptoms. Several studies report a beneficial effect of supplementation of 25(OH)D for musculoskeletal complaints [18–20]. We hypothesized that a substantial proportion of unselected patients from primary care presenting with musculoskeletal complaints may have vitamin D deficiency.

Thus, the aim of the present study was (a) to estimate the prevalence of 25(OH)D deficiency in unselected patients from primary care; (b).to assess a potential association with two typical musculoskeletal symptoms (pain and weakness), fatigue, body mass index, age, intake of Vitamin D, and skin type; and (c) to evaluate seasonal variations (summer and winter) of serum 25(OH) concentrations.

## Methods

### Ethics statement

The study was approved by the local ethics committee (Ethics committee of the canton of Luzern/Switzerland) and participating patients provided their written informed consent.

### Study design

The study used an observational design. In order to optimise the methodological procedure we preceded according to the STROBE (strengthening the reporting of observational studies in epidemiology) guidelines [21]. Blood sample were taken during September 2011 (end of summer) and March 2012 (end of winter) at the end of a consultation.

### Recruiting participants and inclusion criteria

Overall, 16 general practices in the greater agglomeration of the city of Lucerne, Switzerland were approached by the principal investigator and all agreed to participate in the study. All practices are accepting patients without constraints related to the topic of this study. Participating GPs (n = 20) agreed that in all consecutive patients for whom a blood test was ordered for any reason, 25(OH)D concentration, serum calcium and phosphate, alkaline phosphatase and creatinine levels would also be measured. A patient was eligible for the study if: (a) presenting to their GP for any reason either during the month of September or March or both, and (b) a blood test was ordered by the GP. Patients were excluded if one or more of the following were present: (a) inability to provide informed consent or (b) refusal to participate.

### Outcome measures

The principal outcome was serum 25-(OH)D concentration and its potential seasonal variations. The definition of vitamin D deficiency is not consistent in the literature. For most authors 25(OH)D concentration from 25 to 49 nmol/l are defining the state of 25(OH)D deficiency (for some authors deficiency ranges from 25 to 75nmol/l [22]), whereas levels above the threshold concentration of 75 nmol/l are required for optimal bone and muscle health in younger and middle aged adults and prevention of fractures and falls in older adults age 60 years and older [23, 24]. The definition of severe 25(OH)D deficiency varies from levels below 20 to 30 nmol/l, widely accepted is the limit of 25 nmol/l [4] because the risk of osteomalacia in adults (rickets in children) sharply rises with levels below this limit [5]. For the present study we defined 25(OH)D levels of <50 nmol as vitamin D deficiency, and <25 nmol/ as severe vitamin D deficiency. Parathyroid hormone is optimally suppressed above the 25(OH)D threshold concentration of 75 nmol/l[5]. Therefore, we considered this level as the optimal threshold regarding optimal bone and muscle health. Further outcomes were vitamin D deficiency associated symptoms, i.e., muscle weakness, musculoskeletal pain, and fatigue, and associated predictors, i.e., age, body mass index, intake of Vitamin D and skin type.

### Procedure

Eligible patients were approached by the GP during a regular consultation. All patients in whom GPs ordered a blood test were asked whether they agreed that an additional blood tube (serum, gel-separator, Sarstedt) was taken to measure 25(OH)D concentration, serum calcium and phosphate, alkaline phosphatase and creatinine. While waiting for blood sample taking patients had the opportunity to read the study information leaflet and gave written informed consent. Weight and height was measured by the practice nurse prior to taking the blood samples. Patients were asked if they had felt musculoskeletal pain and/or general muscular weakness or if they had perceived being tired during the last four weeks prior to the consultation. The quantification of these three symptoms was assessed by using a visual analogue scale (VAS). Patients were asked to rate their symptoms from 0 (none) to 10 (maximal intensity) on the VAS. Patients completed the questionnaire containing the visual analogue scales in the waiting room of the practice. Baseline characteristics were gathered by the GP from the patient’s medical record. The barcoded blood samples were transported by courier on the same day from the GP’s practice to the laboratory.

### Measuring of Vitamin D

Laboratory tests were performed in the accredited laboratory Bioanalytica Lucerne, Switzerland. After automated aliquoting (Modular Pre-Analytics, Roche, Switzerland), one serum aliquot of each patient was frozen at—20°Celsius for measurement of 25(OH)D in batch later. Measurement of 25(OH)D was performed with an assay (Immunodiagnosticsystems IDS, Frankfurt a. Main, Germany) according to instructions of the manufacturer [25] on an automated microtitre well analyzer (Euroanalyzer 1, Euroimmun, Switzerland). This assay was calibrated against a reference LC-MS/MS method. Briefly, samples were diluted with Biotin-labelled 25(OH)D and incubated in microtitre wells coated with highly 25(OH)D specific sheep antibodies for 2 hours at room temperature. After washing, horseradish peroxidase labelled avidin was added and binds selectively to complexed biotin. After a second washing step, a chromogenic substrat (TMB) was added and the concentration of 25(OH)D calculated as inversely proportional to the intensity of the developing colour read. Interassay and intraassay precision range from 4.6–8.7% and 5.3–6.7%, respectively, depending on the concentration of 25(OH)D in the sample. Cross-reactivity to 25(OH)D3 and 25(OH)D2 was 100% and 75% resp. No significant cross- reactivity to the non hydroxylated D3 und D2 was observed.

Serum creatinine, calcium, phosphate and alkaline phosphatase were measured continuously during daily routine by the Cobas6000 system. The estimated glomerular filtration rate was calculated using the Cockcroft-Gault-formula.

### Statistical analysis

The results are presented as proportions, means, and standard deviations (SDs), unless otherwise specified. To calculate differences between vitamin D deficient and non-deficient patients t-tests were used. To calculate relations between vitamin D concentration and other continuous variables Spearman’s rank correlation tests were used. A p-value of <0.05 was considered to be statistically significant. All data were calculated using the Stata statistical package, version 11.2 (Stata Incorporation, College Station, TX, USA).

## Results

Overall, blood samples were taken in 1844 patients. Of these, 156 patients were excluded from the study due to incomplete data acquisition (e.g. haemolytic blood samples, incomplete questionnaires, lacking data for weight and height). Only six patients explicitly refused to participate (did not give written informed consent). Eventually, 1682 (91.2%) individuals with complete data were included for analysis (776 subjects in September, 906 subjects in March, respectively; S1 Dataset). An overview of patients’ characteristics is given in Table 1. No significant differences between the variables assessed end of summer and end of winter were found (p > 0.05). Very few patients (n = 19, 1.1%) had severe chronic kidney disease with glomerular filtrations rates below 30 ml/min. A minority (n = 13, 6.3%) of patients with serum 25(OH)D concentration below 25 nmol/l (n = 207) showed elevated alkaline phosphatase (>120 Units/l).

**Table 1:** Characteristics of the study populations assessed end of summer (month of September) and end of winter (month of March).

	Summer (n = 776)	Winter (n = 907)
Age (years [±SD; Range])	55.0 (±19.5; 14–94)	56.7 (±18.5; 8–92)
Male (n [%])	300 (38.7)	331 (36.5)
Height (cm [±SD])	168 (±9)	168 (±22)
Weight (kg [±SD])	71.2 (±15.4)	71 (±16)
BMI (kg/m^2^ [±SD])	25.2 (±4.8)	25.2 (±5.2)
Caucasians (n [%])	750 (96.7)	870 (95.9)
GFR (ml/min [±SD])	98.8 (±39.0)	92.4 (±35.1)
Corrected serum calcium (mmol/l [±SD])	2.18 (±0.09)	2.21 (±0.09)
Serum albumin (g/l [±SD])	45.1 (±3.1)	43.7 (±2.9)
Serum alkaline phosphatase (Units/l [±SD])	71.9 (±34.9)	69. (±30.3)
Patients taking vitamin D supplements (n [%])	103 (13.3)	111 (12.2)

No significant differences were found between variables assessed end of summer and end of winter (p > 0.05)

The mean 25(OH)D serum concentration of all participating patients was 47.1 ± 20.4 nmol/l (range 5.0 to 155.7). In the whole study population (n = 1682) serum concentration of 25(OH)D were < 75nmol/l, < 50nmol/l, and < 25nmol/l in 1541 (91.6%), 995 (59.2%), and 207 (12.3%) patients, respectively.

The mean muscle pain and weakness, fatigue, body mass index (BMI), and age in 25(OH)D deficient (< 50nmol/l) and non-deficient (>50 nmol/l) patients are shown in Table 2. 25(OH)D deficient patients reported more intense muscle weakness (visual analogue scale 2.7, 95% confidence interval 2.5 to 2.9) and had a higher body mass index (25.9kg/m2, 25.5 to 26.2) than non-deficient patients (2.5, 2.3 to 2.7; and 24.2, 23.9 to 24.5, respectively). No differences were found for musculoskeletal pain, fatigue, and age.

**Table 2:** Mean muscle pain and weakness, fatigue, body mass index (BMI), and age in 25(OH)D deficient (<50 nmol/l) and non-deficient (>50 nmol/l) patients.

Variable	25(OH)D serum concentration < 50 nmol/l	25(OH)D serum concentration > 50 nmol/l	p-value^a^
Muscle pain (VAS)	2.7 (2.5–2.9)	2.5 (2.3–2.7)	0.13
Muscle weakness (VAS)	1.6 (1.4–1.8)	1.3 (1.1–1.4)	0.02
Fatigue (VAS)	3.6 (3.4–3.8)	3.4 (3.1–3.6)	0.16
BMI (kg/m^2^)	25.9 (25.5–26.2)	24.2 (23.9–24.5)	< 0.001
Age (years)	56.5 (55.3–57.7)	55.5 (54.0–57.0)	0.30

VAS, Visual analogue scale, BMI, body mass index;

^a^t test

Correlations of patients’ muscle pain and weakness, fatigue, BMI, and age with 25(OH)D serum concentrations are shown in Table 3. 25(OH)D concentrations weakly correlated with muscle weakness (Spearman’s rho -0.059, 95% confidence interval -0.107 to -0.011) and body mass index (-0.156, -0.202 to -0.108). Self-reported musculoskeletal pain, and fatigue did not correlate with concentrations. Age did not correlate with concentrations in the overall population, nor in those older than 65 (n = 611, rho = -0.079, p = 0.06), 75 (n = 303, rho = -0.061, p = 0.29), and 80 years (n = 161, rho = -0.122, p = 0.12), respectively.

**Table 3:** Correlations of patients’ muscle pain and weakness, fatigue, body mass index (BMI), and age with 25(OH)D serum concentrations.

Variable	Mean	Correlation with 25(OH)D serum concentration^a^	95% confidence interval	p-value^b^
Muscle pain (VAS)	2.6 ± 3.2	-0.032	-0.080 to 0.016	0.19
Muscle weakness (VAS)	1.45 ± 2.7	-0.059	-0.107 to -0.011	0.02
Fatigue (VAS)	3.5 ± 3.3	-0.027	-0.074 to 0.021	0.28
BMI (kg/m^2^)	25.2 ± 5.0	-0.156	-0.202 to -0.108	< 0.001
Age (years)	56.1 ± 19.1	-0.028	-0.075 to 0.020	0.26

VAS, Visual analogue scale, BMI, body mass index;

^a^ Spearman’s rho,

^b^ Spearman’s rank test

Identical calculations were performed with a cut-off level of 75 nmol/l for defining deficient and non-deficient patients. Using this cut-off value, no differences regarding associations were found.

In total, 214 patients (12.7%) were taking drugs containing 25(OH)D. A majority of subjects (79.8%) took a combination therapy consisting of 500 mg calcium and 400 units of 25(OH)D once or twice daily. The remaining patients were prescribed 25(OH)D alone as a liquid solution (10 drops corresponding to 1000 units of 25(OH)D once daily). Mean 25(OH)D concentrations in patients taking vitamin D containing medication were higher (mean 60.6 ± 22.2 nmol/l) than in patients without medication (44.8 ± 19.2 nmol/l, p < 0.0001), but still below the targeted level of 75 nmol/l. There was no difference in serum calcium levels between those taking vitamin D supplements and those who were not (p = 0.32). Differences of 25(OH)D concentration in patients with skin type [26] I and II (96.3% of study population) compared to individuals with skin types IV and V (47.5 ± 20.5 nmol/l vs. 36.0 ± 17.0 nmol/l, p = 0.0001) were found.

**Fig 1:**
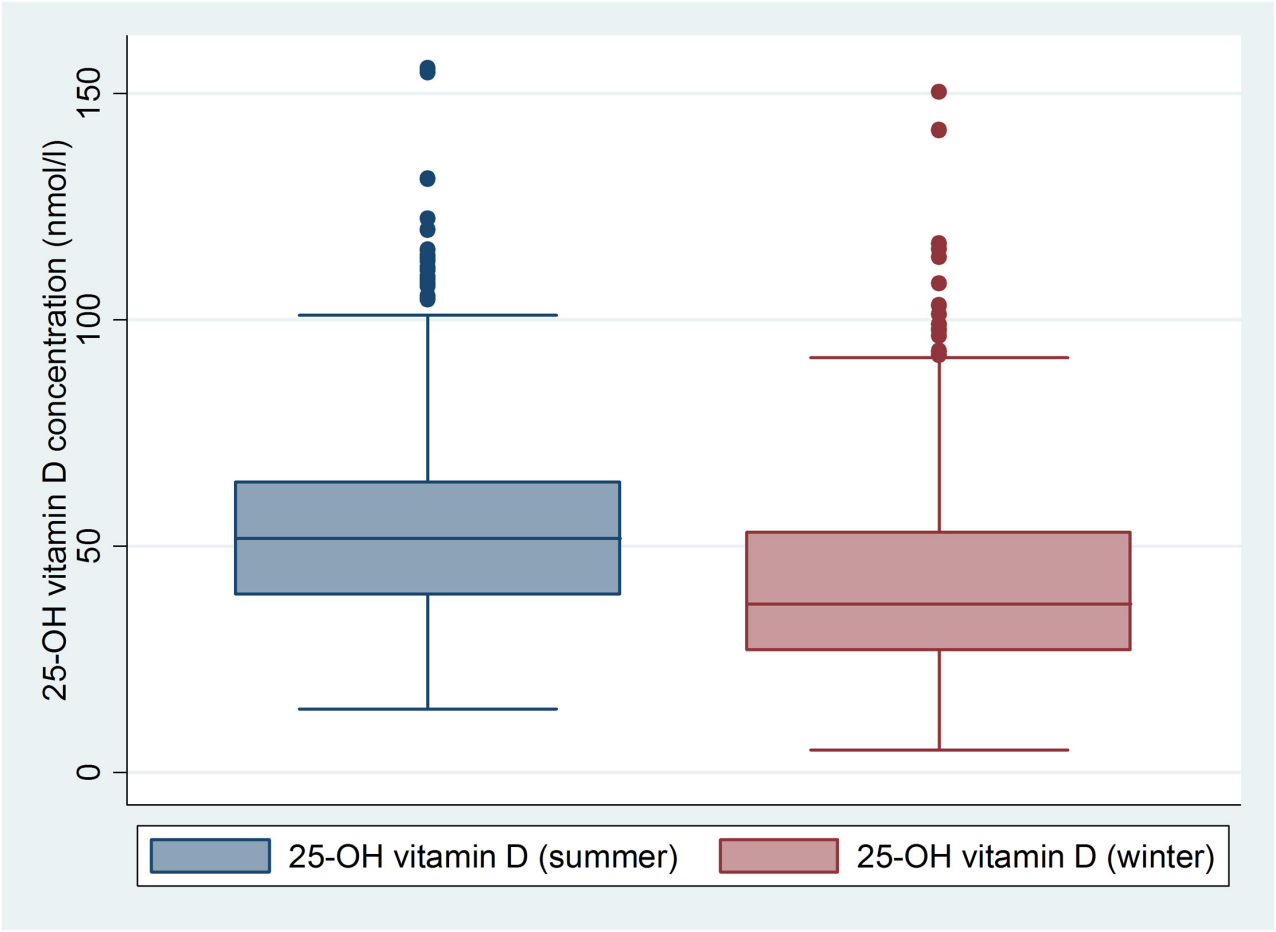
Significant difference of 25-hydroxy 25(OH)D concentrations end of summer and end of winter (53.4 ± 19.9 vs. 41.6 ± 19.3 nmol/l, p<0.0001).

Comparison of 25(OH)D concentration end of summer and end of winter are shown in Fig 1. The seasonal 25(OH)D concentrations differed (53.4 ± 19.9 vs. 41.6 ± 19.3nmol/l, p < 0.0001). At the end of summer, 88.9% of patients (n = 690) had values < 75nmol/l, 350 patients (45.1%) had values < 50nmol/l, and 34 patients (4.4%) had values < 25nmol/l. End of winter, 93.9% (n = 851) of patients were < 75nmol/l, 71.2% (n = 645) < 50nmol/l, and 18% (n = 173) < 25nmol/l, respectively. In a subpopulation of 93 patients blood samples were taken both at the end of summer and the end of winter. In these individuals the seasonal 25(OH)D also differed (56.2 nmol/l vs. 49.4 nmol/l, p = 0.01).

## Discussion

### Summary of findings

Self-reported muscle weakness and high body mass index were associated with lower 25(OH)D levels. A vast majority of the patients studied had 25(OH)D concentrations below the recommended target [23] concentration of 75 nmol/l for optimal bone and muscle health [5].

### Comparison with former publications

The prevalence of patients with 25(OH)D concentrations below target level of 75 nmol/l in our sample is in line with the results of a recent report of a Swiss rheumatology outpatient population (86%) [22] and moderately higher than the 50–70% estimated by an EU report [27]. Our findings confirm the concerns of the EU report from 2010 addressing that vitamin D deficiency is a huge public health issue [27]. Our results also strengthen the need for action to tackle the growing problem of hypovitaminosis D in order to ensure the maintenance of normal bone health and the preservation of normal muscle function in the population. The fact that 12.3% of the study participants had severe 25(OH)D deficiency below 25 nmol/l is also important since severe vitamin D deficiency is considered to be a relevant risk factor for experiencing bone fractures particularly among elderly individuals. In a Swiss study among patients with acute hip fractures over 50% had severe 25(OH)D deficiency [28] and in the European SENECA-study elderly men and women showed severe deficiency in 36% and 47%, respectively [29]. Although the prevalence of severe vitamin D deficiency in our unselected sample might appear low, this finding is particularly relevant from a health economic point of view. More than one out of ten patients in a typical primary care population was exposed to a significantly increased risk of experiencing a relevant fracture due to severe 25(OH)D deficiency. This may substantially affect health care expenditures by generating cost-intensive sequelae such as hospitalizations, functional disability, and potential requirement for permanent nursing home care.

As to be expected, 25(OH)D levels were significantly lower at the end of winter compared to levels end of summer. The differences were present in the whole study population as well as in a subsample of 93 patients in whom 25(OH)D levels were measured in both seasons. These results highlight the pivotal role of decreased sun exposure as the main risk factor for 25(OH)D deficiency, namely the seasonal decrease in winter. Other potential conditions of decreased UVB exposure are represented in higher geographical latitude, dark pigmentation of the skin, recreational behaviour and use of sunscreen [11]. A review of 25(OH)D levels in different regions of the world showed that female sex, higher latitude, winter season, less sunlight exposure, dietary habits, and the absence of 25(OH)D fortification, darker skin pigmentation, and older age are the main factors that are significantly associated with lower 25(OH)D concentration levels [30]. Not surprisingly non-Caucasian study participants showed lower 25-OH-D3 concentration in our study. The capacity of the skin to produce 25(OH)D is considered to decline with age, thus seniors have been reported to have a four times lower capacity than younger adults [14, 31].

An unexpected finding was that we could not document a correlation between 25(OH)D concentration and age. One may argue that other risk factors such as recreational behavior or living in nursing homes—both associated with lack of sun exposure—may play a more relevant role than the age of a person. This might be supported by the fact that we studied ambulant patients who were able to visit a GP and not living in nursing homes.

Obesity (body mass index (BMI) ≥30 kg/m2) is also considered to be a risk factor for 25(OH)D deficiency probably because 25(OH)D is sequestered into fat tissue [32]. In a recent review a weak but significant inverse correlation between serum 25(OH)D concentration and BMI in an adult population has been shown [33]. In our patients from a primary care setting we could confirm this inverse association between body fat and the level of 25(OH)D concentrations.

Our results suggest a correlation between muscle weakness and the degree of 25(OH)D deficiency. In the literature the association between 25(OH)D deficiency and muscular weakness is reported to cause proximal myopathy of the lower extremities, and so increases the risk of falls [34]. Typically, in 25(OH)D deficient patients type II muscle fibres are diminished [2]. Type II muscle fibre are responsible for rapid muscular movements which are crucial in preventing falls. Therefore, a recommendation to screen for 25(OH)D deficiency in patients complaining of muscular weakness seems prudent provided that other typical and treatable causes of muscle weakness such as hypokalemia, hypothyroidism and drugs (steroids and statins) are excluded [35].

Fatigue and musculoskeletal pain are also common complaints in primary care and are reported to be associated with 25(OH)D deficiency [4]. In clinical practice the evaluation of a patient presenting with fatigue is challenging since the differential diagnosis of fatigue is broad. In the subpopulation of patients that were assessed in summer we found that those with 25(OH)D deficiency more frequently reported fatigue than patients without deficiency (p = 0.009). However, in the whole study population (patients assessed in summer and winter) no differences regarding 25(OH)D concentrations were found. This may imply that screening for 25(OH)D deficiency in patients evaluated for fatigue is not the first step, but might be considered in absence of other explanatory causes.

We found no differences between patients with normal 25(OH)D levels compared to individuals with 25(OH)D deficiency in terms of musculoskeletal pain. Patients with vitamin D deficiency may have myalgia arising from vitamin D induced muscle pathology. This issue is multifaceted since vitamin D induced osteomalacia is also associated with bone pain and microfractures, making causal discrimination of the source of pain challenging. Some published evidence on this topic suggests an improvement in non-specific musculoskeletal pain after substituting vitamin D in patients with 25(OH)D deficiency [36–38]. However, this issue is controversial since other studies do not support this association [39, 40]. Only one randomized placebo-controlled trial assessed whether supplementation of vitamin D improves diffuse muscle pain in patients attending community rheumatology clinics[39]. The study was adequately powered to detect a clinically meaningful change in pain score but there was no benefit of treatment, i.e. no improvement in pain scores was seen in either group. Therefore, larger randomized placebo-controlled trials are warranted to evaluate whether vitamin D supplementation has a role in patients with vitamin D deficiency and musculoskeletal pain. There is no evidence to support supplementation for myalgia in people with normal 25(OH)D levels.

### Strengths and limitations

The major strength of this study is the inclusion a typical GP population. The GP practices were typical practices for Switzerland. The current study allows to approximate the prevalence of vitamin D deficiency in GP practices which is of importance for public health interventions. Generalizability of our results to other countries would need to be evaluated. For the purpose of this paper, we stuck to unadjusted analysis dealing with observed associations in GP offices. These associations need to be further strengthened to be considered cause or effect.

### Implications

Our finding might have several implications for clinical practice. First, our data suggest that vitamin D deficiency seems to be a prevalent phenomenon in daily clinical practice not only in winter but also in summer. Second, particularly in (obese) patients presenting with unspecific muscular weakness, measuring vitamin D concentrations might be an option in the diagnostic process since substitution of vitamin D in recommended doses is inexpensive and has few side effects. Third, although there is growing epidemiological evidence suggesting many other beneficial health effects of 25(OH)D concerning risk of certain cancers, cardiovascular, infectious and autoimmune diseases [5, 6], these suggested benefits are not yet proved by large randomized clinical trials. Ongoing projects such as DO-HEALTH study in Europe (www.do-health.eu) and the VITAL study in the US (www.vitalstudy.org) will probably give more insight into the non-musculoskeletal health issues of vitamin D.

### Conclusions

We conclude that in 90% of unselected patients from primary care 25(OH)D concentrations were below recommended targets of 75 nmol/l and two thirds of subjects fulfilled the criteria of vitamin D deficiency (< 50 nmol/l). Self-reported muscle weakness but not fatigue, and musculoskeletal pain were associated with lower serum 25(OH)D levels. Body mass index was inversely correlated with serum vitamin D concentrations whereas unexpectedly no correlation between age and 25(OH)D levels was found. As excepted significant seasonal variations of 25(OH)D concentrations were documented.

## Supporting Information

S1 Dataset Anonymized dataset. (XLSX)
